# High-Resolution Melting Genotyping of *Enterococcus faecium* Based on Multilocus Sequence Typing Derived Single Nucleotide Polymorphisms

**DOI:** 10.1371/journal.pone.0029189

**Published:** 2011-12-16

**Authors:** Steven Y. C. Tong, Shirley Xie, Leisha J. Richardson, Susan A. Ballard, Farshid Dakh, Elizabeth A. Grabsch, M. Lindsay Grayson, Benjamin P. Howden, Paul D. R. Johnson, Philip M. Giffard

**Affiliations:** 1 Menzies School of Health Research, Darwin, Northern Territory, Australia; 2 Department of Infectious Diseases and Microbiology, Austin Health, Heidelberg, Victoria, Australia; Institut Pasteur, France

## Abstract

We have developed a single nucleotide polymorphism (SNP) nucleated high-resolution melting (HRM) technique to genotype *Enterococcus faecium*. Eight SNPs were derived from the *E. faecium* multilocus sequence typing (MLST) database and amplified fragments containing these SNPs were interrogated by HRM. We tested the HRM genotyping scheme on 85 *E. faecium* bloodstream isolates and compared the results with MLST, pulsed-field gel electrophoresis (PFGE) and an allele specific real-time PCR (AS kinetic PCR) SNP typing method. *In silico* analysis based on predicted HRM curves according to the G+C content of each fragment for all 567 sequence types (STs) in the MLST database together with empiric data from the 85 isolates demonstrated that HRM analysis resolves *E. faecium* into 231 “melting types” (MelTs) and provides a Simpson's Index of Diversity (*D*) of 0.991 with respect to MLST. This is a significant improvement on the AS kinetic PCR SNP typing scheme that resolves 61 SNP types with *D* of 0.95. The MelTs were concordant with the known ST of the isolates. For the 85 isolates, there were 13 PFGE patterns, 17 STs, 14 MelTs and eight SNP types. There was excellent concordance between PFGE, MLST and MelTs with Adjusted Rand Indices of PFGE to MelT 0.936 and ST to MelT 0.973. In conclusion, this HRM based method appears rapid and reproducible. The results are concordant with MLST and the MLST based population structure.

## Introduction

*Enterococcus faecium* emerged as a hospital-associated pathogen in the 1990s in the USA, Europe and Australia. The acquisition of resistance to ampicillin and vancomycin and spread of successful vancomycin-resistant clones has resulted in an increasing burden of vancomycin-resistant *E. faecium* (VREfm) as a difficult to treat nosocomial infection [1].

Pulsed-field gel electrophoresis (PFGE) has been considered the reference method for tracking outbreaks due to its high discriminatory power [2]. However PFGE remains difficult to standardize between laboratories, and the high genomic plasticity of *E. faecium* means that using PFGE data to track strains over long time periods and to infer the large scale population structure can be problematic [1]. In contrast, multilocus sequence typing (MLST) provides portable genotyping data that unambiguously reveal the population structure and long-term patterns of dissemination [3]. However, MLST is laborious and expensive. Furthermore, whole genome sequencing costs have significantly decreased and may in the future make it increasingly difficult to justify MLST.

However, in the meantime, a rapid, simple and low cost *E. faecium* genotyping method that provides results concordant with MLST would be useful for *E. faecium* monitoring and the initial investigation of suspected VREfm outbreaks. In this study a typing method was developed based upon the identification and high-resolution melting (HRM) interrogation of DNA fragments containing resolution-optimized sets of SNPs derived from the *E. faecium* MLST database. The use of such SNP sets in allele specific real-time PCR (AS kinetic PCR) based bacterial genotyping has been previously described [4]–[6]. The adaptation of this approach to HRM technology is a recently reported innovation [7], [8]. HRM analysis of PCR fragments incorporates information from not only the key SNP of interest but also neighboring SNPs. This additional information can result in a surprisingly improved discriminatory power [7]. We compared and validated the HRM method against PFGE, MLST and an AS kinetic PCR method [6] using a set of 85 well characterized *E. faecium* bloodstream isolates.

## Methods

### *Enterococcus faecium* isolates

A collection of 85 *E. faecium* blood culture isolates previously described and typed by MLST were included in this study [9]. The collection comprised 34 VREfm and 51 vancomycin-susceptible (VSEfm) isolates, encompassed 17 STs and reflected the epidemiology of *E. faecium* over a 12 year period at a single Australian institution. The isolates were collected as part of an infection control quality improvement project and formal approval by the local ethics committee was not required.

### DNA extraction

DNA was prepared using the DNeasy Tissue Kit (Qiagen Pty Ltd., Victoria, Australia) and the modified protocol for isolation of genomic DNA from Gram-positive bacteria as described in the manufacturer's instructions.

### Pulsed-field gel electrophoresis

PFGE of *SmaI*-digested genomic DNA agarose plugs was performed as previously described [10]. Gels were analyzed with GelCompar II version 3.5 (Applied Maths NV, Sint-Martens-Latem, Belgium) using Dice Coefficient and represented by unweighted pair group method with arithmetic mean (UPGMA) with 0.75% optimization and 1% tolerance. Isolates with similarity of 80% or more were considered a clonal cluster.

### Selection of SNPs

Resolution optimized SNPs from the concatenated *E. faecium* MLST database at http://efaecium.mlst.net/ were derived using the program ‘Minimum SNPs’ [11]. SNPs that did not change the %G+C content (C↔G, A↔T) were excluded as these are not usually detected by HRM. Primers were designed using Primer Express 2.0.0 (Applied Biosystems) and obtained from Proligo (Mulgrave, Victoria, Australia). Sequences of primers are given in **Table 1**.

**Table 1:** The eight fragments: Primers, fragment sizes and normalization regions.

Region name	Primer sequence (5′–3′)		Fragment Size (bp)	Normalization regions (°C)
*atpA*95	F	CCTTGGCGATTTCGAGTCC	180	79–8085–86
	R	CCTGTTTGCATTGGTTCGTTAAC		
*atpA*239	F	GTTAACGAACCAATGCAAACAGG	108	76–7785–86
	R	ATACAGATCATATCTTGACCTTTTTGG		
*atpA*485	F	TCTACAGTTCGTACACAAGTTGAAAC	87	76–7784–85
	R	CACCCATTGCAGTACCAGCA		
*ddl*204	F	GAAGGTTCTTTGCTTTATCCGATG	79	75–7682–83
	R	CGAGAATCATACTGATAGGCTGTTG		
*ddl*456	F	GTGAAGTCGTAAAAGACGTAGC	97	74–7581–82
	R	TAACATCGTGTAAGCTAACTTCGC		
*purK*460	F	GGTGGCAGGAAATGGTCAAG	107	78–7985–86
	R	GCAATCACACGGGCAATACG		
*pstS*95	F	TGTATTTGCAGAAGAAAGAGACG	141	75–7680–81
	R	TGATCTTTCCCGCCAACTT		
*pstS*452	F	CAAGCACTAAGTATCGACGGTGTAG	79	74–7581–82
	R	TCTAAGAATTTTTGGACTTCAGGG		

### HRM-based typing

Reactions were performed on a RotorGene 6000 device (Corbett, Mortlake, NSW, Australia). Reactions volumes comprised 5 µL 2× Platinum® SYBR® Green qPCR SuperMix-UDG (Invitrogen Life Technologies, Mt Waverley, Australia), 1 µM of each primer and 2 µL of DNA (1∶10 dilution) in a final volume of 10 µL. The temperature cycling parameters were: 95°C for 2 min; 30 cycles of 95°C for 5 s, 55°C for 20 s; followed by 72°C for 2 min and 50°C for 30 secs. Amplification was followed by HRM (72–88°C) increasing by 0.1°C at each step. Runs were performed in laboratories in two separate institutions by multiple operators and results were completely concordant and reproducible. Normalization temperatures for analysis of each fragment were standardized (**Table 1**). The results for the real-time PCR experiments were analysed using the Rotorgene 6000 software v 1.7 (Corbett) and data was exported to Microsoft Excel 2007 and GraphPad Prism v5.01 (GraphPad Software, Inc, California, USA). Three known isolates were included in each run for calibration purposes. To determine ‘across run comparisons’ the inter-run difference in the melting temperature (T_m_) for these three isolates was determined, and the mean T_m_ difference to the closest 0.1°C was used to calibrate the other samples. Use of the three known control isolates in each run also facilitated the accurate assignment of HRM alleles (see below).

### HRMType® software

We used a previously described Stata/IC 10.1 (StatCorp, College Station, Texas, USA) “do_file” named HRMType® to assemble a predicted key for translating between MLST and multilocus HRM data [7]. This procedure is based on the premise that the T_m_ of each amplified fragment is in large part dependent on the absolute G+C content. HRMType® converts each MLST database-specified variant of each HRM fragment into a predicted HRM allele on the basis of G+C content. For example, for the *atpA*95 fragment, in the current *E. faecium* MLST database, the numbers of G+Cs are 79, 80, 82, 83, 84, 85. HRMType® then converts each known ST into a predicted multilocus HRM type that is termed a “Melting Type” (MelT), and displays each ST with a corresponding MelT. Our convention is that the HRM alleles are numbered according to the absolute number of G+C residues and the MelTs are numbered sequentially commencing with ST1 corresponding to MelT 1. If during experimental work a fragment (e.g., *atpA*239 for ST178) does not produce a curve as predicted according to G+C content, the unpredicted curve can be assigned a new HRM allele number. For this fragment (e.g., *atpA*239), this new HRM allele number is assigned for all other STs that have the identical sequence in the fragment (e.g., there are 20 other STs with the same *atpA*239 fragment sequence as ST178). HRMType® also calculates the cumulative Simpson's Index of Diversity (*D*) [12] in comparison to MLST as each fragment is added to the set.

### *In silico* SNP genotyping

We adapted HRMType® to allow the input of the specific eight SNPs used by Rathnayake et al. [6] and produce a translation key between STs and the concatenated sequence of SNPs, which we have termed ‘SNP types’. For each of the 85 isolates, SNP types were determined by *in silico* analysis of the MLST sequence.

### Determination of the concordance between HRM typing and the MLST defined E. faecium population structure

The Adjusted Rand Indices (ARI) were calculated using Comparing Partitions [13]. To generate the goeBURST diagram we used the algorithms and software as described by Francisco et al. [14] and found at http://goeburst.phyloviz.net/. This allows for the overlaying of the MelTs onto a snapshot of the MLST derived population structure of *E. faecium*.

## Results

### Identification of a resolution optimized set of SNPs

A set of eight SNPs within the MLST loci was identified on the basis of maximized *D*. The SNPs were (locus name followed by nucleotide position within that locus): *atpA* 95, *atpA* 239, *atpA* 485, *ddl* 204, *ddl* 456, *purK* 460, *pstS* 95 and *pstS* 452. Amplified fragments incorporating these SNPs ranged in size from 79 to 180 bp (**Table 1**). *In silico* analysis based on predicted T_m_ differences suggested that these eight fragments would partition the 567 sequence types (STs) currently in the MLST database into 230 Melting Types (MelTs) with a *D* of 0.991, where the MLST database is regarded as providing *D* = 1. This indicates a probability of 0.991 that any two randomly selected STs will be discriminated according to their MelT.

Notably, the resolution optimized SNPs were identified in only four of the seven MLST loci. Examination of the MLST loci determined that these are the four most polymorphic loci with the largest number of alleles and individually they also provide the most discriminatory power compared to MLST. Specifically, the number of alleles (n) and *D* for the seven loci are (in order of discriminatory power): *atpA* (n = 58, *D* = 0.911), *ddl* (n = 45, *D* = 0.843), *purK* (n = 49, *D* = 0.841), *pstS* (n = 66, *D* = 0.724), *gdh* (n = 42, *D* = 0.643), *adk* (n = 25, *D* = 0.557), *gyd* (n = 27, *D* = 0.498).

### High-resolution melting curve analysis

**Figure 1:**
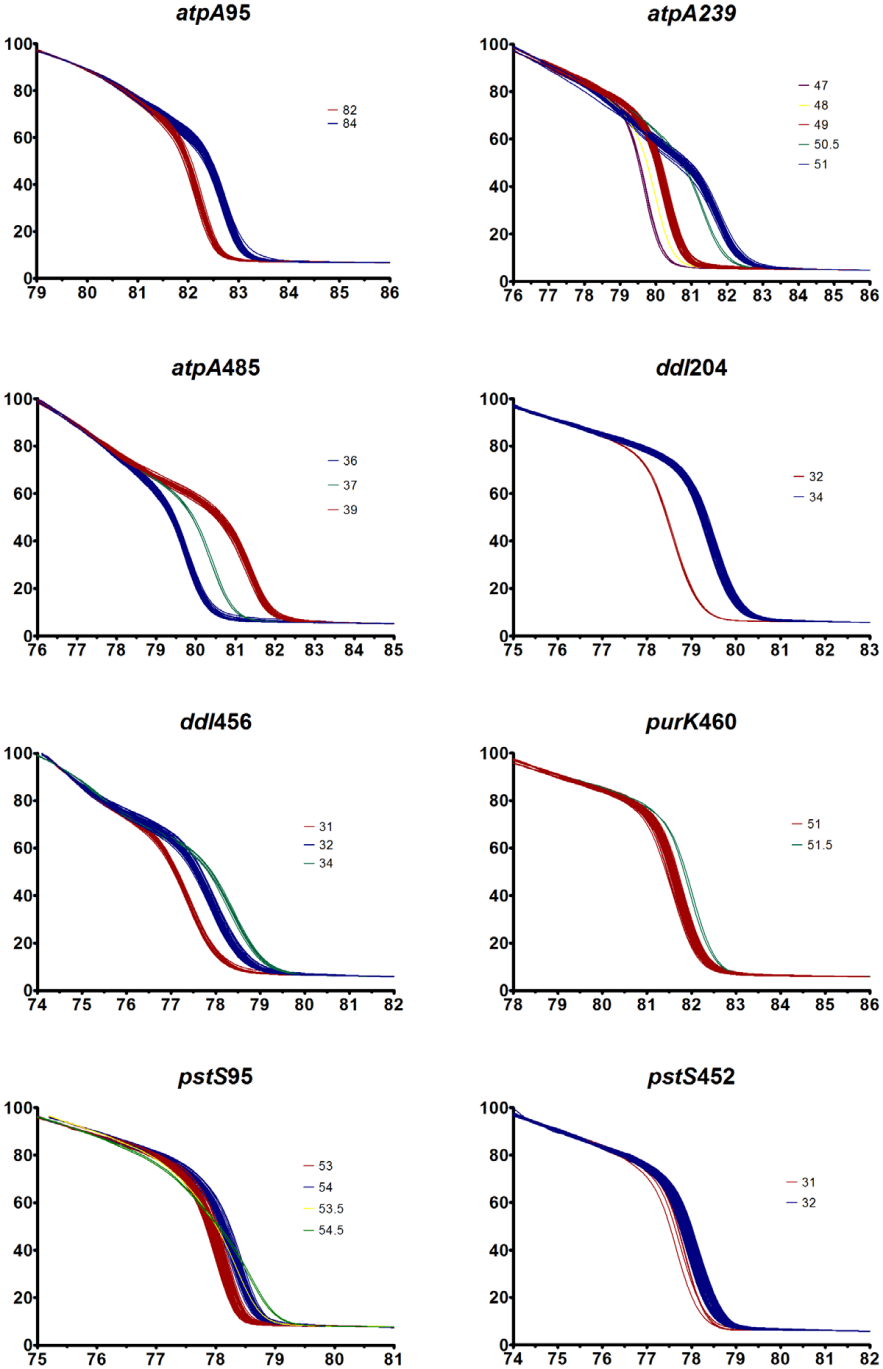
High-resolution melting curves for the eight fragments. The normalized fluoresence (y-axis) is plotted against temperature in °C (x-axis) and shows the curves for 85 *E. faecium* isolates. Each graph is labelled with the amplified SNP and combines two HRM runs that have been calibrated against each other; different curves are represented by a different color and accordingly labelled.

**Figure 2:**
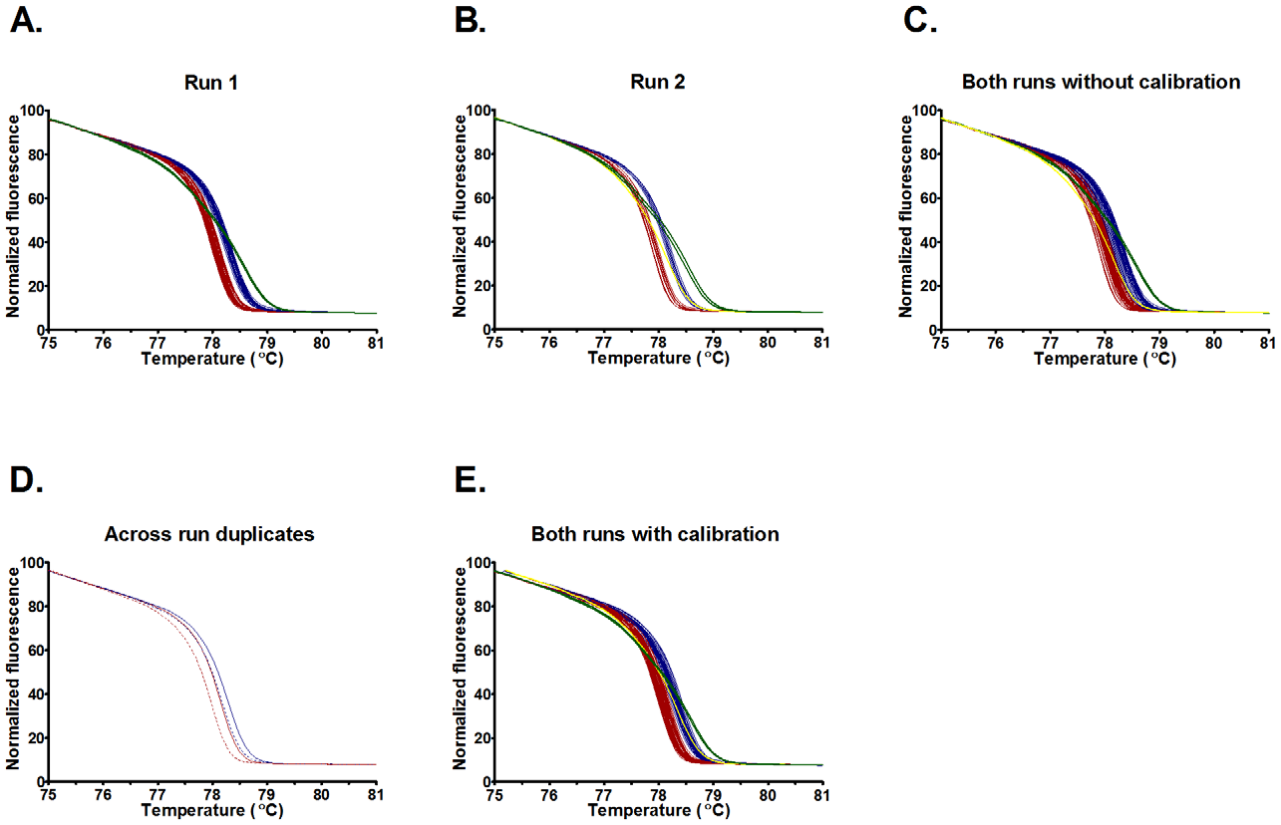
High-resolution melting curves for pstS95. The graphs represent (**A and B**) HRM curves for two separate runs demonstrating discrimination of curves 53 (red) and 54 (blue) when each run is considered separately; (**C**) raw data from both runs combined without calibration, demonstrating an inability to discriminate curves 53 and 54; (**D**) raw data from two isolates included in both runs (solid red and dashed red lines for curve 53 isolate and solid blue and dashed blue for curve 54 isolate) demonstrating a shift in the curves when runs are compared without callibration; (**E**) data from both runs combined with calibration demonstrating discrimination of curves 53 and 54.

**Figure 3:**
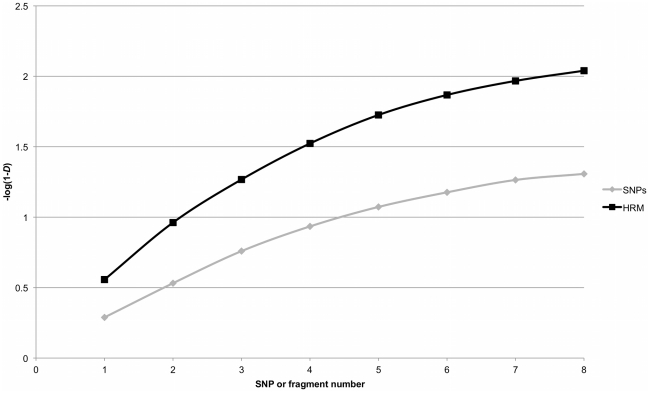
Comparison of HRM and SNP methods. Cumulative Simpson's Index of Diversity on a log scale for high-resolution melting and single nucleotide polymorphism methods against all 567 s multilocus sequence types.

We performed HRM genotyping for these eight fragments on 85 *E. faecium* isolates [9]. The HRM curves from each of the eight fragments for all 85 isolates are displayed in **Figure 1**. The displayed curves have been calibrated for inter-run variability, however we found that, with the exception of fragment *pstS*95, the raw data from the curves could have been exported without calibration and the curves correctly assigned. For fragment *pstS*95 (**Figure 2**), curves 53 and 54 were discriminated in each individual run, but when raw data from the two runs were combined, these curves could not be discriminated. An inter-run shift of 0.2°C for the calibrating isolates was evident; following calibration, curves 53 and 54 were discriminated in the combined dataset. The HRM curves produced were in accordance with G+C content, with minor exceptions for three isolates. For two ST178 isolates unpredicted curves were seen at *atpA*239, *purK*460, and *pstS*95; and for an ST495 isolate there was an unpredicted curve at *pstS*95 and the predicted curve 50 at *purK*460 could not be reliably discriminated from curve 51. The key generated by HRMType® was adjusted to take into account the unpredicted curves and converted into an Excel based key to facilitate ongoing interpretation (**Results S1**). Following this adjustment, there were 231 MelTs for the 567 STs in the MLST database and a *D* of 0.991. In comparison, *in silico* SNP genotyping with the previously described eight SNPs provides 61 SNP types and a *D* of 0.951. The cumulative resolving power of HRM typing compared to SNP genotyping was found to be clearly superior; in fact, using four HRM fragments provides significantly higher resolution than all eight SNPs (**Figure 3**).

### Comparison of HRM typing with MLST, PFGE and SNP genotyping

**Figure 4:**
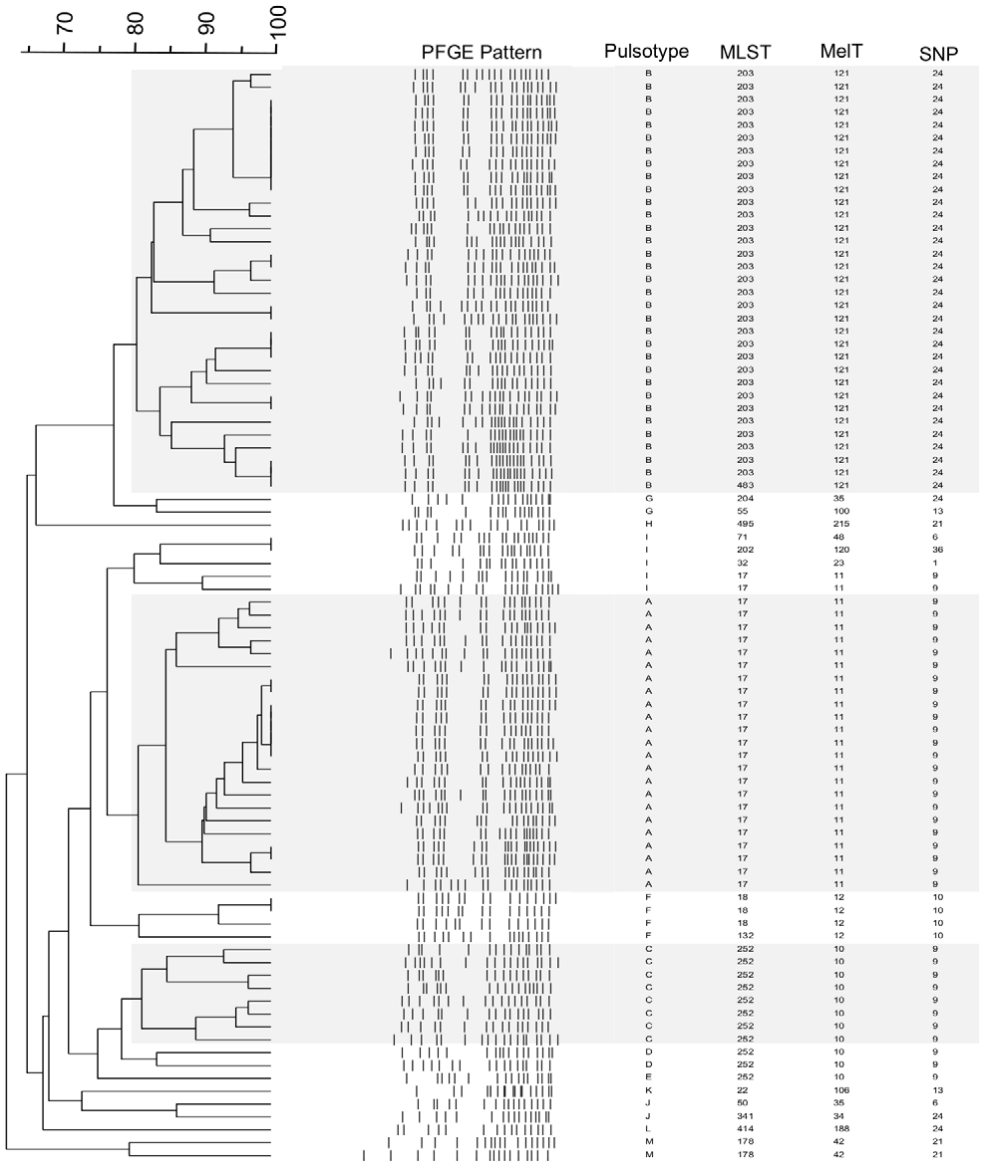
PFGE dendrogram of 85 isolates. Dendrogram of pulsed-field gel electrophoresis clusters (major clusters highlighted by grey shading) with corresponding multilocus sequence types (MLST), melting types (MelTs) and *in silico* determined single nucleotide polymorphism types (SNP types). PFGE clusters are based on 80% similarity.

We compared the discriminatory power and concordance of genotyping results between HRM typing, MLST, PFGE and SNP genotyping for the 85 isolates (**Figure 4**; **Results S2**). The 85 isolates were partitioned into 13 PFGE patterns, 17 STs, 14 MelTs and 8 SNP types with *D* values of 0.77, 0.76, 0.75 and 0.64 respectively. The ARI for PFGE to ST was 0.908, PFGE to MelT 0.936, and ST to MelT 0.973, indicating excellent concordance between these three methods.

### Correlation with population structure as determined by goeBURST of *E. faecium* MLST

**Figure 5:**
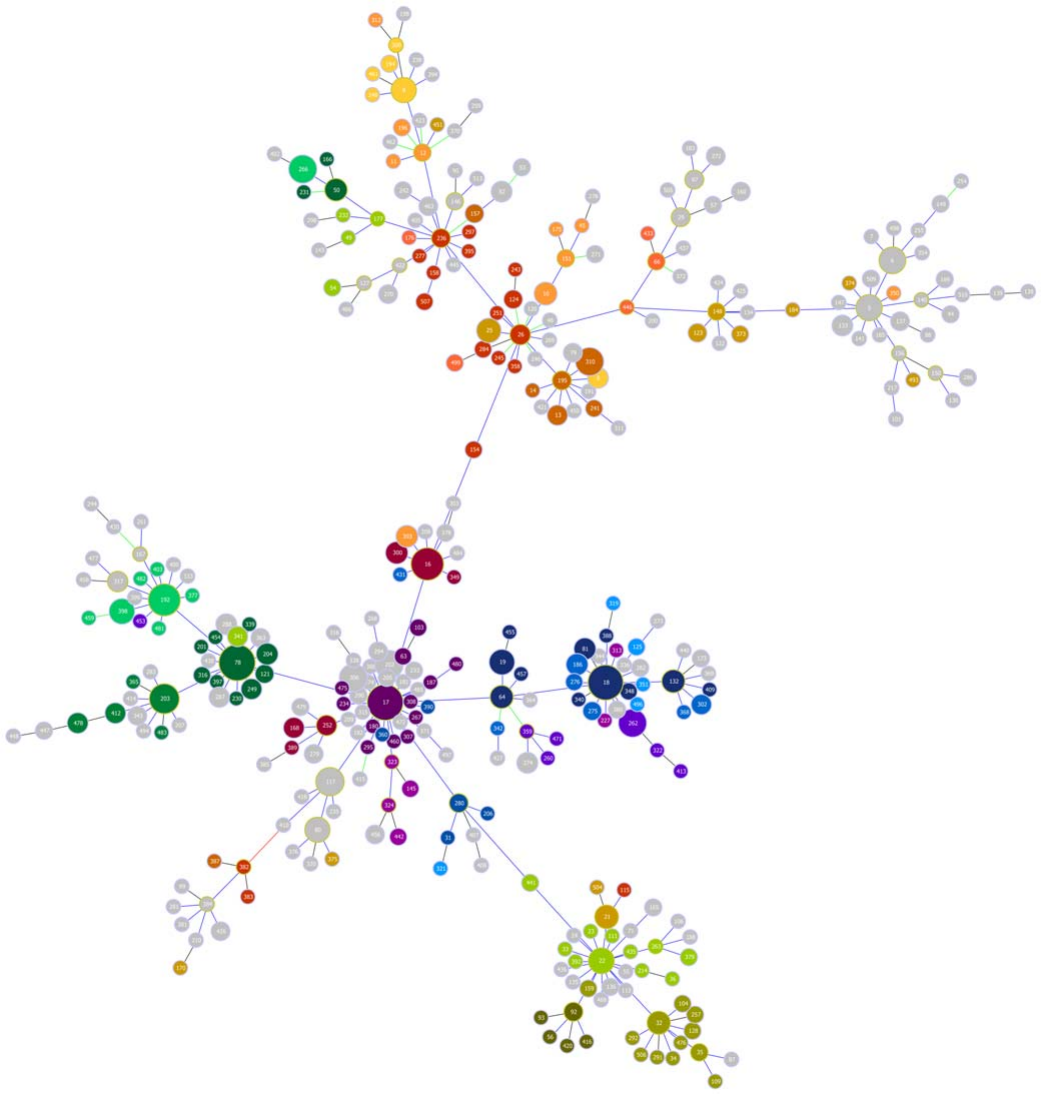
goeBURST diagram of the *Enteroccocus faecium* clonal complex 17 population. goeBURST diagram of the *Enteroccocus faecium* clonal complex 17 population from the multilocus sequence type (ST) database with superimposed melting types (MelTs). The top 26 MelTs (each with ≥5 associated STs) are represented by different colors. Other STs in grey are represented by smaller MelTs that each have <5 associated STs. STs are indicated by numbers in the circles, the size of each circle represents the relative abundance of that ST, and lines indicate single locus variants.

We produced a population snapshot from the 1457 *E. faecium* isolates in the MLST database using goeBURST and superimposed the 26 MelTs that incorporated five or more STs onto the CC17 cluster (**Figure 5**). This demonstrated that the key subgroup founders within CC17 are discriminated from each other by HRM genotyping. There were some anomalies revealed by this analysis as demonstrated by MelTs of the same color not all clustering together. For example, ST50 does not cluster with ST78 on the goeBURST diagram but does share the same MelT 35. This can be explained in that ST50 and ST78 are actually double-locus-variants (DLVs) of one another, but the goeBURST algorithm does not cluster them together. Similarly ST313 and ST227 may be single-locus-variants (SLVs), DLVs or triple-locus-variants (TLVs) of other members of MelT 98 (ST145, 323, 324, 442) and thus be closely related but not be clustered together on goeBURST; and the STs within MelT 105 (ST186, 275, 276, 302, 342, 368 and 431) are all SLVs or DLVs of each other but are not clustered together on goeBURST.

## Discussion

We have developed and validated an HRM based method for genotyping *E. faecium* that can be performed at low cost and high throughput. Our analysis indicates that the use of eight HRM reactions resolves the existing 567 STs into 231 MelTs with a *D* of 0.991 compared to all 567 STs. As validated against a collection of 85 *E. faecium* isolates, HRM genotyping has excellent correlation with and similar resolving power to MLST and PFGE.

There are a number of limitations to the HRM method. First, it is not usually possible to discriminate fragments with the same G+C content but of a different sequence using HRM. Thus, the discriminatory power of HRM typing will be less than that of full MLST. Second, novel MLST alleles may be missed if a mutation does not change the G+C content or if a mutation is found outside of the eight fragments being interrogated. Third, as the MLST database is updated with new STs being deposited, the HRMType® translation key will need to be updated. For this purpose, frequently updated keys can be found at http://menzies.edu.au/research/tropical-and-emerging-infectious-disease/bacterial-genotyping. Fourth, we have validated this method on a collection of local isolates that may not be representative of the global collection of STs, although the collection does contain major clones such as ST17, 18, 22, 32, and 203.

The HRM typing method shows a high level of concordance with the *E. faecium* population structure, as inferred by goeBURST analysis. Instances of non-concordance are very likely due to the limitations of algorithms such as eBURST [15] and goeBURST [14]. These limitations are particularly acute in recombinogenic species such as *E. faecium* [16] and care must be taken in the interpretation of such diagrams. Specifically, a large interlinked complex such as CC17 may simply reflect intensive sampling of part of the population by MLST, and it is possible for quite unrelated STs to be part of such complexes, provided they are linked by several intermediate STs. Conversely, a large interlinked complex in a recombinogenic species may define so many essentially equivalent topologies that there will be instances of closely related STs being separated by anomalously large numbers of intermediate STs. Consistent with this, we determined that STs that are not resolved by HRM typing, and yet are distant on the goeBURST diagram, are actually much more closely related than the goeBURST diagram indicates. Additionally, the large interlinked CC17 likely incorporates several instances of *E. faecium* clonal radiation of diversification, with the identified founders being ST17, ST18, ST22, ST26, ST78 and ST192 [1]. The HRM method resolves all of these STs. It was therefore concluded that the concordance of population structure and HRM typing is close to as good as it can be, given the finite resolving power of HRM typing, and the considerable inherent difficulties in inferring and depicting the population structures of recombinogenic bacterial species. Given that the HRM method is based on SNPs derived from MLST, any limitations of MLST in depicting the population structure of *E. faecium* will also be present with HRM typing.

With recombinogenic bacterial species, the maximum resolving power that may be obtained from a given number of SNPs is high because linkage between the allelic states of the SNPs is reduced or obliterated by horizontal transfer of chromosome fragments, thus making it possible for resolving power to increase logarithmically rather than linearly as SNPs are added to a typing set [8]. Even if only the “nucleating” SNPs are taken into account, the SNPs defined many more than eight genotypes, thus indicating that *E. faecium* recombination has greatly increased the resolving power conferred by a given number of SNPs. Interestingly, these SNPs may be clustered in certain loci, and for *E. faecium* the unbiased algorithm used to identify SNPs to maximize resolving power determined these SNPs to be in only four of the seven MLST loci. Not surprisingly, these are the four most polymorphic and discriminatory MLST loci. Use of SNPs from four, rather than seven, MLST loci is further justified in that results from HRM typing are concordant with MLST. Our methods for identifying regions optimized for genotyping and interpreting the data with reference to databases of sequence variation are not restricted to the HRM platform or MLST databases. The methods could be adapted to identify fragments containing resolution optimized SNPs across a genome and to generate a key for other platforms such as with base composition analysis using PCR/electron-spray mass spectrometry [17].

Depending on the monetary, time and capillary sequencing resources available, MLST can be resource intensive for many laboratories. HRM typing utilizes eight PCR reactions with little downstream processing and is thus significantly cheaper than MLST, which requires an initial seven PCR reactions followed by capillary sequencing and analysis. Although less discriminatory than MLST, HRM typing can be used as an initial genotyping scheme to rapidly screen isolates at low cost while providing results that are concordant with MLST. For example, it can be used to identify globally recognized and endemic strains of *E. faecium* and only where indicated, such as during a local outbreak, would PFGE, MLST or whole genome sequencing be required. Even then HRM is a useful tool to focus and reduce the requirement for these more highly discriminating methods. Although MLST continues to be widely used and whole genome analysis using next generation sequencing technologies is becoming the gold standard genotyping methodology (and will probably replace MLST), we feel that there continues to be an important role for rapid, low cost methodologies that make use of existing instrumentation present in many less resourced laboratories.

## Supporting Information

Results S1 **Melting type and multilocus sequence type conversion key.**
(XLS)

Results S2 **Results for HRM typing, MLST, PFGE and SNP genotyping for 85 isolates.**
(XLS)
